# Prevalence of and associated factors for sleep disorders in patients with amyotrophic lateral sclerosis: a systematic review and meta-analysis

**DOI:** 10.3389/fneur.2026.1889217

**Published:** 2026-08-31

**Authors:** Genping Xie, Yupeng Guo, Zhuo Wang

**Affiliations:** 1 College of Basic Medical Sciences, China Three Gorges University, Yichang, China; 2 The Second Clinical Medical College of China Three Gorges University, Yichang, China; 3 College of Medicine and Health Sciences, China Three Gorges University, Yichang, China

**Keywords:** amyotrophic lateral sclerosis, associated factors, meta-analysis, prevalence, sleep disorders

## Abstract

**Background:**

Sleep disorders stand as prevalent non-motor symptoms in individuals with amyotrophic lateral sclerosis (ALS) and may be linked to respiratory muscle weakness, neurodegenerative changes, and disruption of sleep architecture. Existing studies have reported considerable variability in the prevalence of sleep disorders in ALS patients, and findings regarding associated factors remain inconsistent. Therefore, this study intended to integrate the existing evidence through a meta-analysis to further clarify the prevalence of sleep disorders and their clinical relevant factors.

**Methods:**

PubMed, Embase, Web of Science, and the Cochrane Library were searched from database inception to January 10, 2026. Cross-sectional, cohort, and case–control studies involving clinically diagnosed adult patients with ALS were included. A random-effects model was used to estimate the pooled prevalence of sleep disorders. Subgroup analyses were performed according to sex, site of onset, age, disease duration, body mass index (BMI), sleep disorder category based on the International Classification of Sleep Disorders, Third Edition, Text Revision (ICSD-3-TR), sleep-related treatment status, and assessment method (polysomnography [PSG] versus questionnaire-based assessment).

**Results:**

A total of 31 studies involving 2,762 patients with ALS were included. The pooled prevalence of sleep disorders was 50% (95% confidence interval [CI], 45–56%). Patients with sleep disorders were significantly older than those without sleep disorders (mean difference [MD] = 3.72 years, 95% CI, 2.39–5.05), whereas no significant associations were observed for sex, body mass index (BMI), disease duration, or site of onset. The prevalence did not differ significantly across the ICSD-3-TR categories (*p* = 0.7367), whereas questionnaire-based studies yielded a significantly higher pooled prevalence than PSG-based studies (*p* = 0.0367). Sensitivity analyses confirmed the robustness of the findings.

**Conclusion:**

Approximately half of ALS patients have sleep disorders. Age may be related to the occurrence of sleep disorders, whereas no substantial relations were identified for sex, BMI, disease duration, or site of onset. Routine screening and early intervention for sleep issues should be strengthened in clinical management. Large-scale, multicenter prospective studies are still needed to further clarify the underlying mechanisms and clinical implications.

**Systematic review registration:**

The publicly accessible registration URL is: https://www.crd.york.ac.uk/PROSPERO/view/CRD420261287352. The systematic review was registered with PROSPERO (registration number: CRD420261287352).

## 1 Background

Amyotrophic lateral sclerosis (ALS) stands as a progressive, fatal neurodegenerative disorder characterized by progressive degeneration and loss of upper and lower motor neurons in the cerebral cortex, brainstem, and spinal cord (1). As the disease progresses, patients gradually develop muscle weakness, muscle atrophy, dysarthria, and dysphagia, and ultimately most die of respiratory failure (2). Epidemiological studies have indicated that the global annual incidence of ALS is about 1–2 per 1,00,000 persons and the prevalence is approximately 4–6 per 1,00,000 persons (3, 4). Although recent progress has been made in genetics and molecular mechanisms, effective treatments that significantly slow disease progression are still lacking, and most patients die within three to 5 years after symptom onset (1, 2). Therefore, ALS not only severely affects individuals’ quality of life but also imposes a heavy burden on families and healthcare systems.

Traditionally, ALS was considered to predominantly involve the motor system. Nevertheless, an increasing number of investigations indicate that it is essentially a neurodegenerative disorder involving multiple systems. Besides motor impairments, individuals may exhibit non-motor symptoms like cognitive decline, mood disorders, autonomic dysfunction, and sleep disorders (5–7). Among these, sleep disorders represent one of the most common and clinically significant non-motor manifestations. They may appear early in the disease course and present as insomnia, sleep-disordered breathing, nocturnal hypoventilation, restless legs syndrome (RLS), and circadian rhythm disturbances (8–10). A recent systematic review and meta-analysis have found that RLS is more prevalent in patients with ALS than in healthy controls. RLS as an important sleep-related comorbidity may further impair sleep and quality of life in this population (11). These abnormalities not only affect nocturnal sleep quality but also lead to daytime sleepiness, fatigue, and mood disturbances, further reducing quality of life (10, 12, 13). The occurrence of sleep disorders in ALS patients may be driven by multiple pathophysiological mechanisms. As the disease progresses, the diaphragm and other respiratory muscles become progressively impaired, making patients more susceptible to nocturnal hypoventilation and carbon dioxide retention during sleep, thereby leading to frequent arousals and disruption of sleep architecture (14–16). Bulbar muscle dysfunction can reduce upper airway stability and increase the risk of sleep-related breathing disorders (10). ALS-related neurodegenerative changes may also involve the brainstem, thalamus, and other central structures involved in sleep regulation, thereby interfering with normal sleep–wake rhythms (17, 18). Factors like pain, spasticity, anxiety, and depression may further aggravate sleep disorders (10, 19). Moreover, sleep apnea and nocturnal respiratory abnormalities are common in ALS and may be closely linked to disease progression and prognosis (20, 21).

However, although investigations on sleep disorders in ALS patients have gradually increased in recent years, existing evidence suggests that sleep disorders are common in ALS patients and may be correlated with reduced quality of life, impaired daytime function, and respiratory-related complications (13, 22, 23). Existing evidence is primarily derived from single-center, small-sample observational studies, and there are differences among studies in terms of study populations, disease stages, and sleep assessment methods, limiting the representativeness and generalizability of individual study findings (24–26). Furthermore, the results of investigations on clinical factors related to sleep disorders in individuals with ALS have not yet been systematically integrated.

Based on this, the current study integrated existing research through a systematic review and meta-analysis. It intended to estimate the overall prevalence of sleep disorders in individuals with ALS and to ascertain associated clinical factors, thereby providing evidence for early identification and clinical management.

## 2 Methods

The current study was implemented as per the Preferred Reporting Items for Systematic Reviews and Meta-Analyses (PRISMA) 2020 guidelines. The protocol was registered in the International Prospective Register of Systematic Reviews (PROSPERO) (registration number: CRD420261287352) (27).

### 2.1 Search strategy

PubMed, Embase, Cochrane, and Web of Science were retrieved from the inception until January 10, 2026. The search strategy combined medical subject headings (MeSH) and free-text terms, and “amyotrophic lateral sclerosis (ALS)” and “sleep” were used as the core concepts. In addition, relevant synonyms and expanded terms like “dyssomnias” and “insomnia” were also incorporated to improve the comprehensiveness of search. No restrictions were imposed on language or publication type during search. The detailed search strategies for each database are presented in the supplementary materials (Supplementary Table S1).

### 2.2 Eligibility criteria and study selection

The inclusion criteria of the current study were outlined below: clinically diagnosed ALS patients aged ≥18 years were included, covering different stages of disease progression. Studies were required to present at least one of the following extractable data: the prevalence of sleep disorders in ALS patients, or data that could be used to calculate this prevalence; comparative data on demographic or clinical characteristics between ALS patients with and without sleep disorders, like age, sex, body mass index (BMI), disease duration, and site of onset; or comparative data on sleep-related parameters between bulbar-onset and non-bulbar-onset ALS patients, like total sleep time (TST), sleep efficiency (SE), percentage of rapid eye movement sleep (REM sleep, %), apnea-hypopnea index (AHI), percentage of deep sleep stage (N3, %), and respiratory disturbance index (RDI). Study designs encompassed cross-sectional, observational cohort (only baseline data included), and case–control studies.

The exclusion criteria were outlined below: reviews, systematic reviews, meta-analyses, case studies, guidelines, letters, and conference abstracts; studies without sleep disorder-related outcome measures or with data unextractable; ALS patients specifically recruited from sleep clinics (e.g., those already diagnosed with insomnia or obstructive sleep apnea) excluded to avoid selection bias.

All records were independently screened by two investigators (G. X. and Y. G.) against titles, abstracts, and full texts. A third investigator (Z. W.) carried out data aggregation and cross-checking. Divergences were addressed through discussion. When necessary, a decision was made by a third investigator.

### 2.3 Data acquisition

Two investigators (G. X. and Y. G.) independently acquired data utilizing a pre-designed standardized Excel form and cross-checked the results. Divergences were addressed through discussion, and when necessary, a decision was made by a third investigator (Z. W.) (27). The acquired data encompassed: first author, publication year, country/region, study design, sample size, mean age, sex ratio, BMI, disease duration, site of onset, ALS diagnostic criteria, as well as the prevalence of sleep disorders and relevant clinical variables.

### 2.4 Risk of bias assessment

The risk of bias of the included studies was independently assessed by two investigators and the results were verified by a third investigator. For cross-sectional and observational cohort studies, the quality assessment tool for observational cohort and cross-sectional studies recommended by the National Heart, Lung, and Blood Institute (NHLBI) of the National Institutes of Health (NIH) was used (28). This tool contained 14 items and primarily evaluated bias in selection, information/measurement, confounding, temporal order, observer, dropout, and statistical power. Regarding case–control studies, the NIH-recommended quality assessment tool for case–control studies was adopted (28). It contained 12 items and mainly assessed bias in selection, information, recall, observer, confounding, and statistical power. Both tools classified the overall quality of studies into three categories: good, fair, and poor.

### 2.5 Data synthesis

To better characterize the spectrum of sleep disorders evaluated across the included studies, we reviewed and classified the primary sleep disorder or sleep-related outcome reported in each study according to the International Classification of Sleep Disorders, Third Edition, Text Revision (ICSD-3-TR), where sufficient information was available. Studies with extractable prevalence data were subsequently grouped according to the corresponding ICSD-3-TR category for subgroup meta-analysis. Prevalence estimates were extracted according to the study-specific definitions reported by the original authors, and no common questionnaire or PSG threshold was retrospectively applied. Studies were also classified as questionnaire-based or polysomnography (PSG)-based according to the method used to assess the selected prevalence outcome. In addition, an exploratory subgroup analysis was conducted based on study-level reported sleep-related treatment status. Because the reported intervention was not necessarily received by all participants and individual-level treatment data were generally unavailable, this analysis was not intended to assess treatment effects. All statistical analyses were implemented utilizing R 4.2.2. First, a meta-analysis was implemented on the overall prevalence of sleep disorders in ALS patients. Heterogeneity across studies was appraised leveraging the *i*^2^ statistic and the *Q* test. For *i*^2^ > 50% or *p* < 0.05, marked heterogeneity was considered, and a random-effects model (DerSimonian–Laird method) was adopted for pooled analysis. Otherwise, a fixed-effects model was applied (29**–**31).

Second, comparative analyses of the prevalence of sleep disorders were performed across different subgroups. Pooled prevalences were calculated for subgroups defined by sex (male, female) and site of onset (bulbar onset, non-bulbar onset). Moreover, between-group difference tests were applied to compare prevalence differences among the subgroups.

Third, to ascertain potential factors linked to sleep disorders, we compared variations in demographic and clinical characteristics between ALS patients with and without sleep disorders, encompassing age, disease duration, and BMI. In addition, variations in sleep-related parameters between bulbar-onset and non-bulbar-onset ALS patients were compared, involving TST, SE, REM sleep, %, AHI, N3, %, and RDI. Continuous variables were depicted as mean difference (MD) and 95% confidence interval (95% CI).

Sensitivity analysis was implemented leveraging the leave-one-out method to evaluate the robustness of the pooled results. All statistical tests were two-sided, and *p* < 0.05 was deemed statistically significant.

## 3 Results

### 3.1 Results of study selection

**Figure 1:**
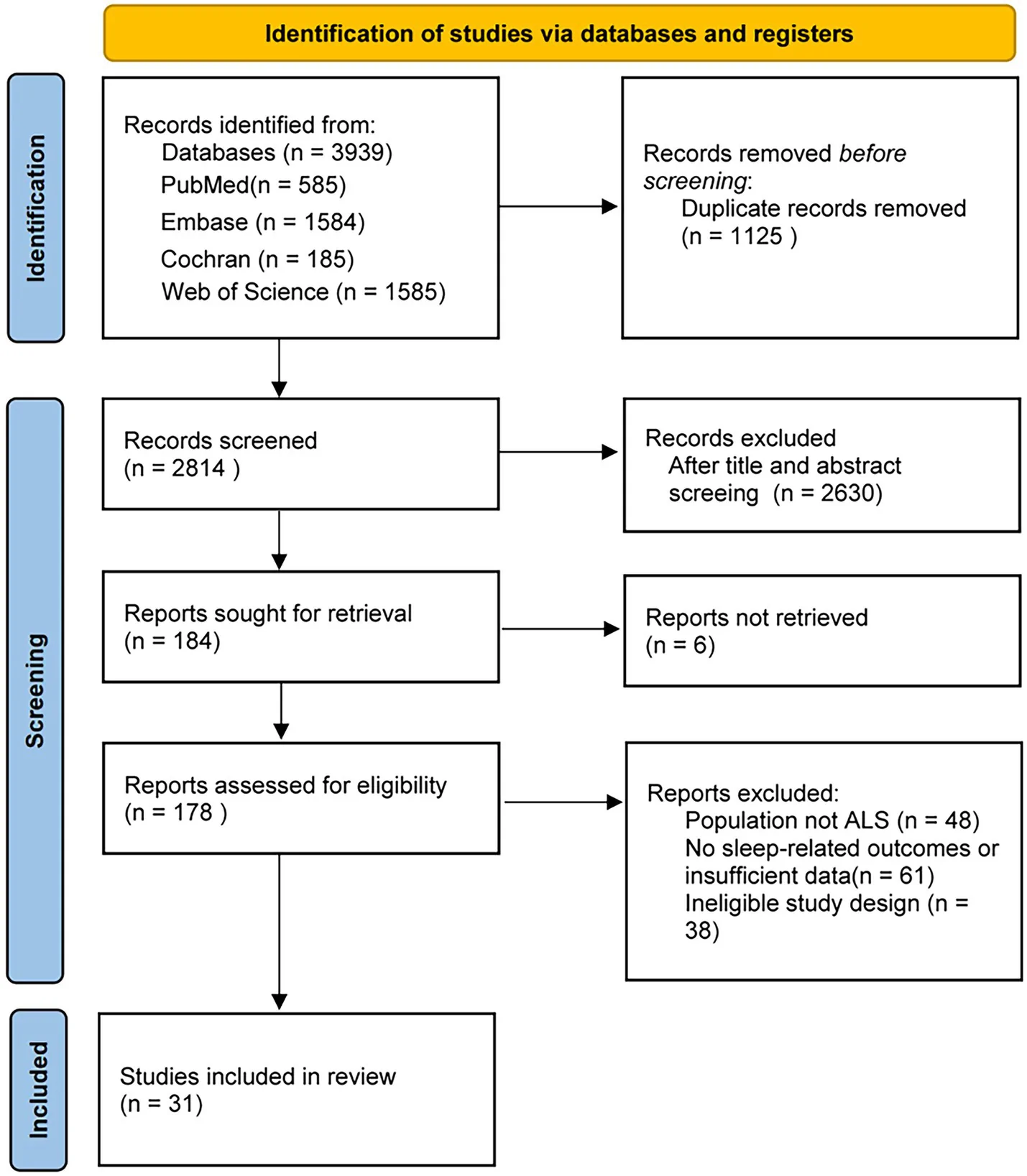
PRISMA flow diagram of study selection.

As per the PRISMA 2020 guidelines, the study selection process is detailed in Figure 1. Totally 3,939 records were initially identified (585: PubMed, 1,584: Embase, 185: Cochrane Library, 1,585: Web of Science). After the elimination of 1,125 duplicate records, 2,814 records were screened against titles and abstracts. Among them, 2,630 were excluded as irrelevant to the research topic. Subsequently, 184 full-text articles were evaluated for eligibility. Among them, 6 were eliminated due to unavailability of full texts. After full-text screening, another 147 articles were removed as a result of non-ALS study populations (*n* = 48), no sleep-related outcomes reported or data unextractable (*n* = 61), and study design not meeting the inclusion criteria (*n* = 38). Finally, 31 studies involving 2,762 ALS patients were incorporated.

### 3.2 Basic characteristics and risk of bias assessment

The included studies were published between 1,991 and 2,025, involving cross-sectional, cohort, and case–control studies (Table 1). The studies covered a wide range of regions, mainly from China, the USA, Italy, Germany, Japan, India, and Portugal. The mean age of ALS patients ranged from approximately 51–68 years, and the majority of studies had a higher proportion of males than females (49.3–80.0%). Among studies that reported BMI, the mean BMI was mostly varied between 23 and 26 kg/m^2^. The proportion of bulbar onset differed substantially across studies, ranging from approximately 14.3 to 83.0%. The reported prevalence of sleep disorders across studies was 14.3–80.8%, and the disease duration was approximately 9.0–58.8 months. The included studies covered multiple ICSD-3-TR sleep disorder categories, primarily insomnia disorders and sleep-related breathing disorders (Supplementary Table S2). Among the 29 included cross-sectional or observational cohort studies, 17 were identified as good, 11 as fair, and one as poor. The study rated as poor had the following methodological issues: unclear description of inclusion/exclusion criteria, a small sample size and no estimation for sample size, insufficient adjustment for potential confounders, incomplete description of outcome or exposure measurement methods, and missing key methodological information in the reported data. Among the two case–control studies, one was determined as good and one as fair. Overall, the majority of the included studies were of good or fair quality, indicating the low to moderate overall risk (Supplementary Table S3 and Supplementary Table S4).

**Table 1:** Basic characteristics of included studies.

Study	Country	Study design	Sample size	Age, years	Male,%	BMI,kg/m^2^	Bulbar onset,%	Sleep disorders,%	Disease duration, months
Jiang 2025	China	Cross-sectional	266	54	61.28	NR	15.41	66.16	17.44
Carratù 2011	Italy	Cohort	31	63.6 ± 8.7	58.06	27.3 ± 4.26	29.03	32.25	11.4 ± 7.6
Kim 2011	South Korea	Cohort	26	53.92 ± 9.83	73.08	NR	NR	80.77	24.51 ± 8.10
Chowdhury 2021	India	Cross-sectional	50	51.28 ± 9.41	80	NR	38	46	13.92
Boentert 2017	Germany	Cross-sectional	250	63.4 ± 10.9	50.8	24.2	33.6	45.6	26.6 ± 19.6
Engel 2021	Germany	Cohort	158	63.9 ± 10.4	54.4	24.8	32.3	61.39	33.4
Boentert 2015	Germany	Cohort	65	63.1 ± 11.7	55.4	25.0 ± 4.3	33.84	53.85	28.2 ± 20.3
Santos 2003	Brazil & Italy	Cross-sectional	114	54.1 ± 11.5	71.9	NR	22.8	30.7	27.6 ± 16.5
Quaranta 2017	Italy	Cohort	42	65.05 ± 8.02	64.3	22.74 ± 2.27	21.42	47.62	26.39 ± 18.83
Reyhani 2020	Turkey	Cohort	73	58.0 ± 9.9	49.3	25.6 ± 4.8	45.2	67.12	58.8 ± 30
Silva 2024	Portugal	Cohort	35	67.86 ± 13.26	68.6	25.94 ± 4.39	NR	37.14	NR
Goudarzi 2023	Iran	Cross-sectional	92	56.5 ± 12.4	74	25.4 ± 5.2	83	43.48	24.0 ± 17.2
GAY 1991	USA	Cohort	21	58.5	76.19	NR	NR	14.29	20.5 ± 16.1
Coco 2012	Italy	Cross-sectional	91	61.26 ± 10.1	60.44	NR	26.4	57.14	24.9 ± 15.4
Zhang 2025	China	Cross-sectional	69	54.88 ± 10.91	63.8	23.63 ± 2.89	14.49	55.07	10.27 ± 3.85
Hirayama 2022	Japan	Cross-sectional	33	60.8 ± 11.2	57.6	NR	NR	48.48	NR
Dors 2019	Germany	Cohort	58	61	51.7	NR	32.8	70.69	19.5
Shojaie 2023	UK	Cross-sectional	506	NR	NR	NR	NR	61.86	NR
Bote 2020	Spain	Cohort	76	60.3 ± 14.4	52.6	NR	36.8	34.21	NR
David 1997	USA	Cross-sectional	17	55	70.59	NR	NR	17.65	NR
Diaz-Abad 2018	USA	Cross-sectional	43	63.8 ± 11.5	53	25.1 ± 3.7	35	62.79	NR
Coco 2016	Italy	Cross-sectional	41	60	17	24 ± 5.9	29.27	53.66	15
Tsara 2010	Greece	Cross-sectional	28	NR	NR	NR	35.7	67.86	11.3 ± 10.5
Bourke 2001	USA	Cross-sectional	23	58	69.6	NR	NR	39.13	28
Kimura 1999	Japan	Cross-sectional	18	57.64 ± 8	61.11	21.37 ± 3.55	61.11	17.65	24.39 ± 16.07
Li 2023a	China	Cross-sectional	56	58.45 ± 8.22	60.71	23.06 ± 1.92	33.93	46.43	25 ± 15.4
Panda 2018	India	Cross-sectional	40	59.00 ± 7.75	57.5	NR	30	70	18
Li 2023b	China	Cohort	63	57.56 ± 10.66	68.3	NR	34.9	38.1	9.0 ± 4.4
Coco 2021	Italy	Case–control	100	59.9 ± 12	60	NR	27	59	25 ± 15.4
Sun 2020	China	Case–control	204	53.5 ± 9.9	55.88	23.4 ± 3.1	30	57.84	12
Tavares 2025	Portugal	Cross-sectional	74	64.39 ± 12.21	59.5	26.66 ± 3.76	NR	60.81	NR

### 3.3 Summary of findings

#### 3.3.1 Overall prevalence of sleep disorders in ALS patients

**Figure 2:**
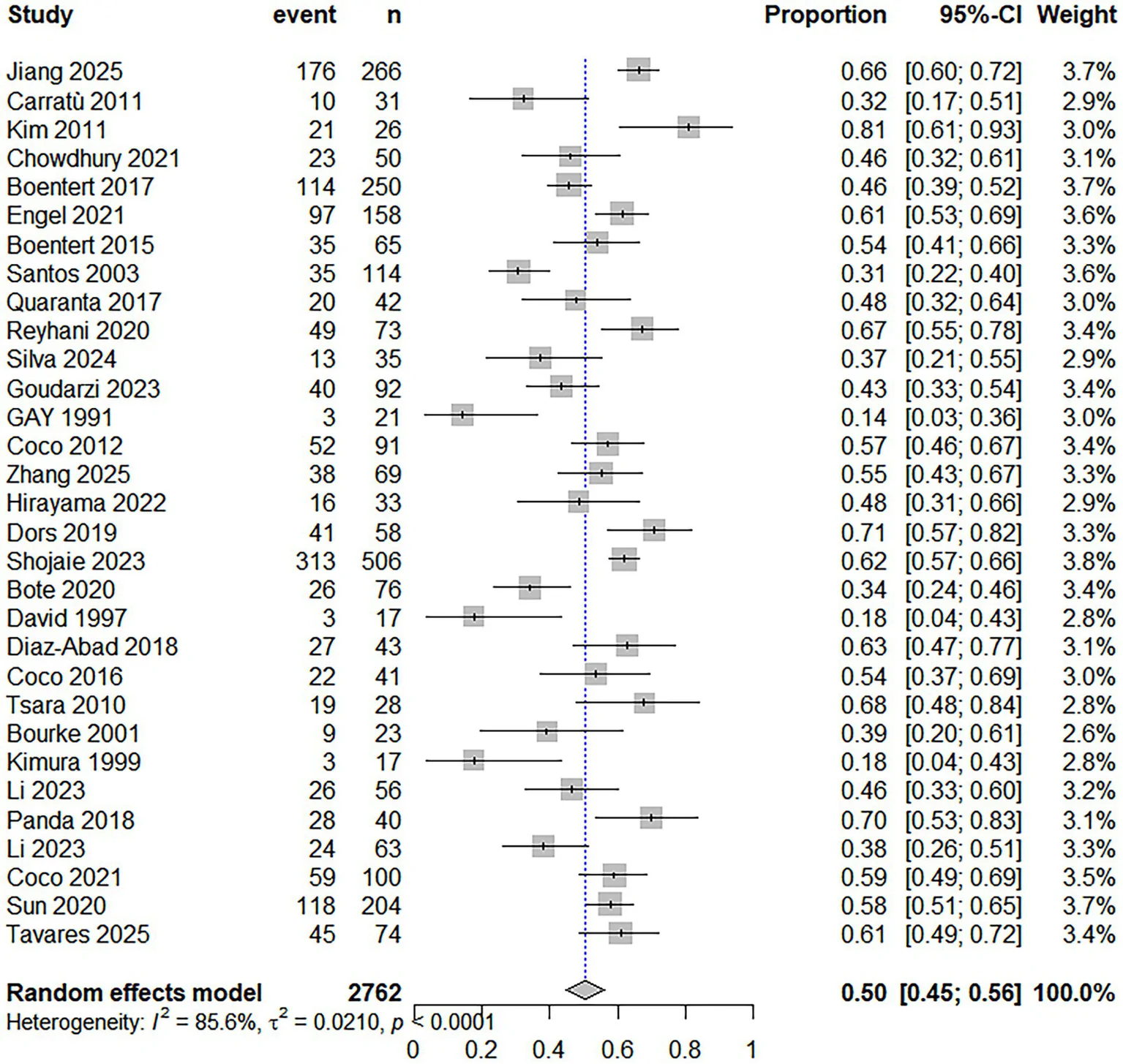
Prevalence of sleep disorders in individuals with ALS.

Totally 31 studies involving 2,762 ALS patients were incorporated in the current study, and a meta-analysis was carried out on the overall prevalence of sleep disorders. The reported prevalence of sleep disorders varied substantially across the included studies, ranging from 14.3% (Gay et al.) to 80.8% (Kim et al.). Detailed study-specific prevalence estimates are provided in Table 1 and Figure 2. Due to substantial heterogeneity across studies (*i*^2^ = 85.6%, *p* < 0.0001), a random-effects model was adopted for pooled analysis. The overall pooled prevalence of sleep disorders in ALS patients was 50% (95% CI: 45–56%). This result indicated that approximately half of ALS patients had sleep disorders of varying degrees. However, given the high heterogeneity, the estimated prevalence should be interpreted cautiously (Figure 2). Sensitivity analysis revealed that the findings were robust (Supplementary Figure S1).

#### 3.3.2 Subgroup analysis by sex

**Figure 3:**
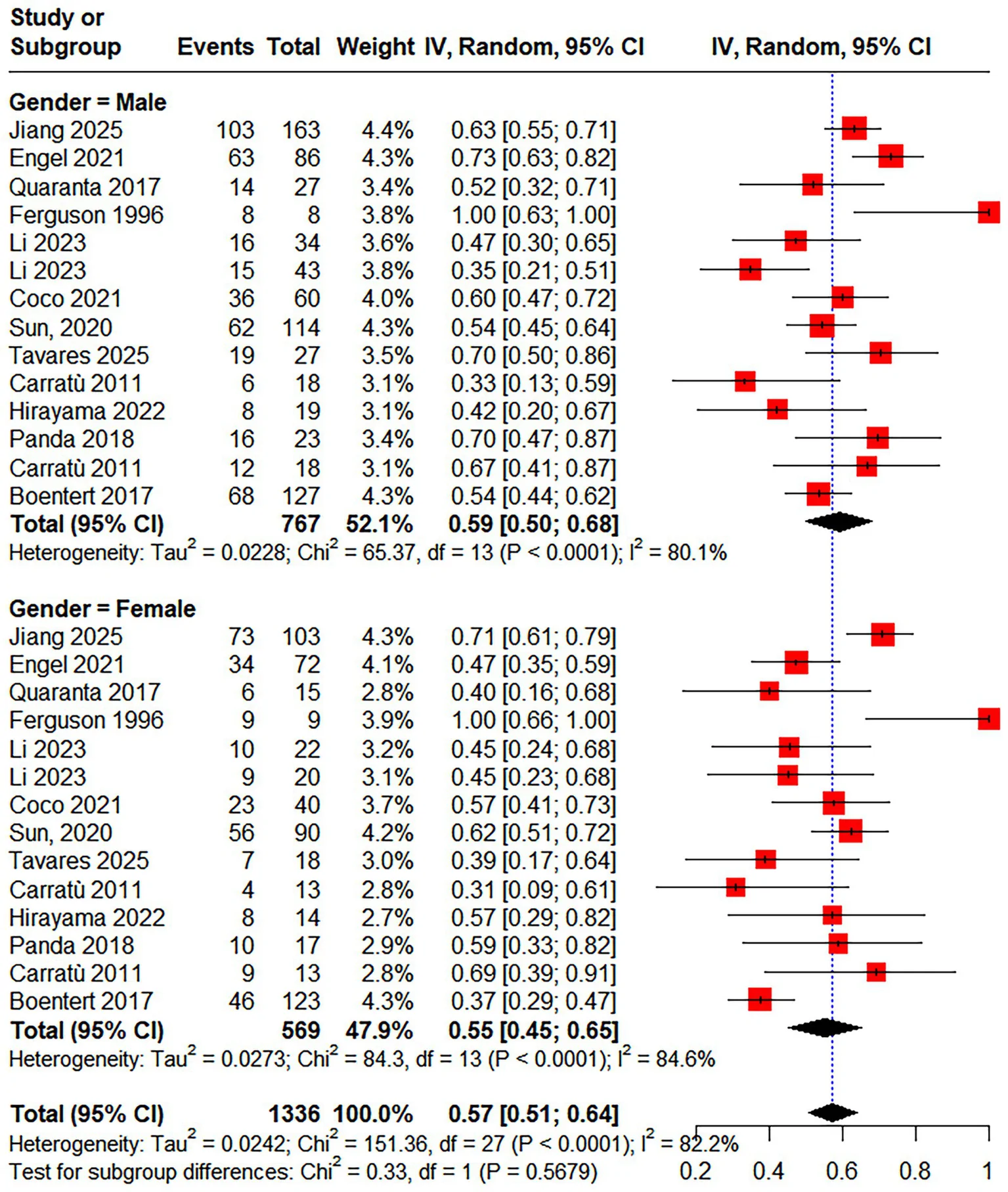
Sex differences in sleep disorders in ALS patients.

According to the results of the subgroup analysis by sex, 28 studies comprising 1,336 ALS patients (767 males and 569 females) were incorporated. The pooled analysis uncovered that the prevalence of sleep disorders was 59% (95% CI: 50–68%) in male patients and 55% (95% CI: 45–65%) in female patients. The variation between subgroups was not statistically significant (*χ*^2^ = 0.33, df = 1, *p* = 0.5679). This result indicated that no substantial relation between sex and the occurrence of sleep disorders in ALS patients was observed (Figure 3).

#### 3.3.3 Subgroup analysis by the site of onset

**Figure 4:**
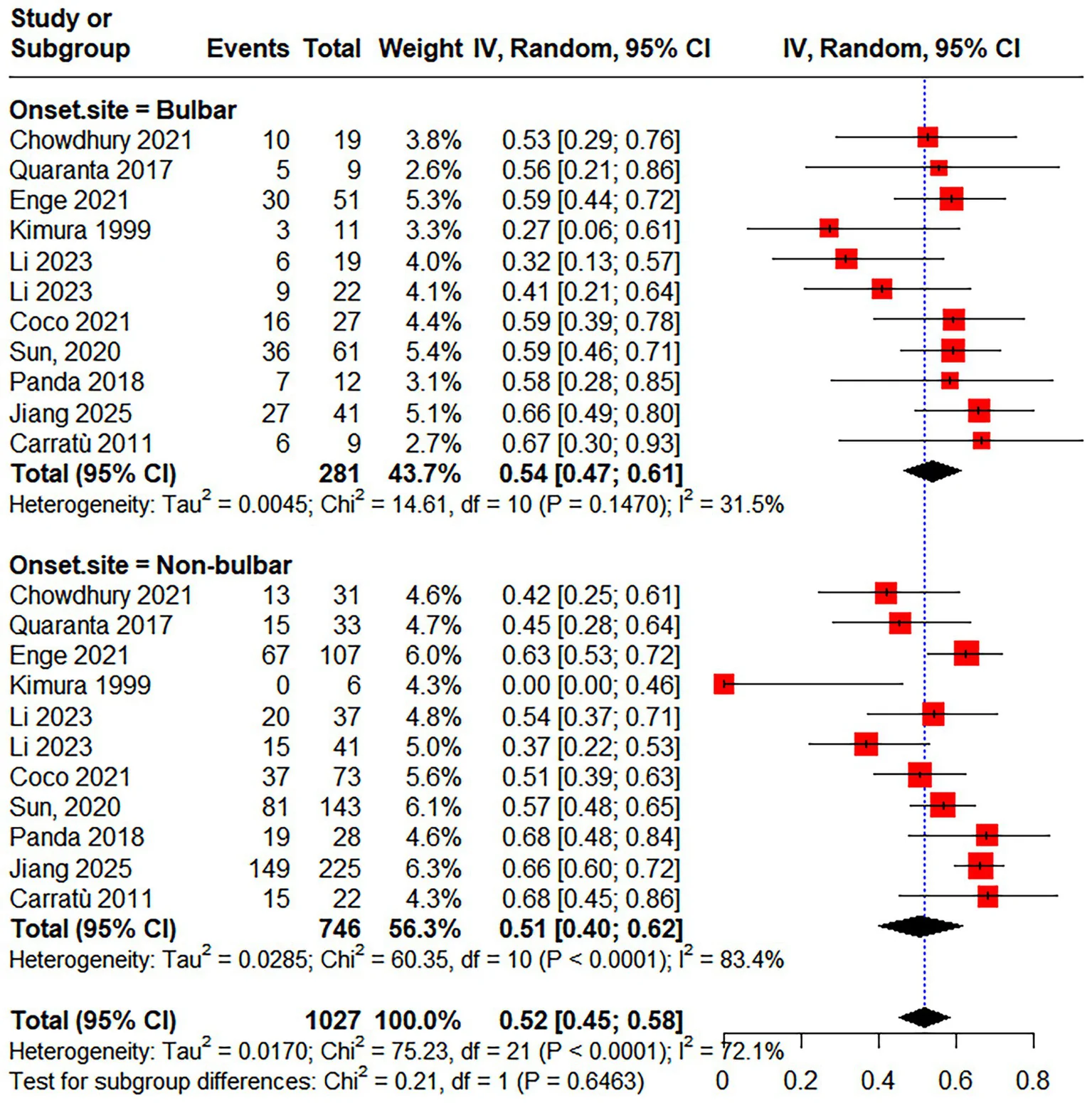
Differences in the site of onset of sleep disorders in individuals with ALS.

According to the site of onset, individuals were stratified into bulbar-onset and non-bulbar-onset subgroups. The bulbar-onset subgroup included 11 studies with 281 individuals, and the pooled prevalence of sleep disorders was 54% (95% CI: 47–61%), with low heterogeneity (*i*^2^ = 31.5%, *p* = 0.147). The non-bulbar-onset subgroup involved 11 studies with 746 individuals, and the pooled prevalence of sleep disorders was 51% (95% CI: 40–62%), with high heterogeneity (*i*^2^ = 83.4%, *p* < 0.001). The variation between subgroups was not statistically significant (*χ*^2^ = 0.21, df = 1, *p* = 0.6463). These results suggested that no marked association was identified between the site of onset and the prevalence of sleep disorders (Figure 4).

#### 3.3.4 Age differences between individuals with and without sleep disorders

**Figure 5:**
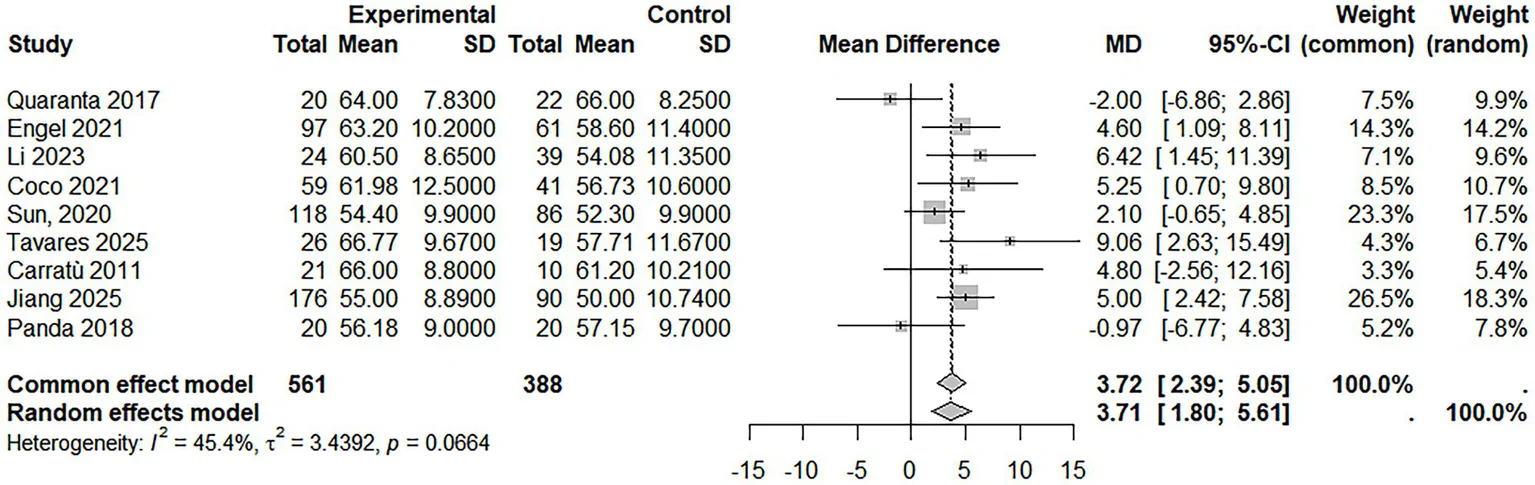
Age differences between ALS patients with and without sleep disorders.

Totally nine studies were incorporated, encompassing 561 ALS patients with sleep disorders and 388 ALS patients without sleep disorders. The heterogeneity across studies was moderate (*i*^2^ = 45.4%, *p* = 0.066), and a fixed-effects model was therefore adopted for analysis. The results demonstrated that the mean age of individuals with sleep disorders was substantially higher than that of individuals without sleep disorders, with a pooled MD of 3.72 years (95% CI: 2.39–5.05) (Figure 5).

#### 3.3.5 Differences in disease duration between individuals with and without sleep disorders

**Figure 6:**
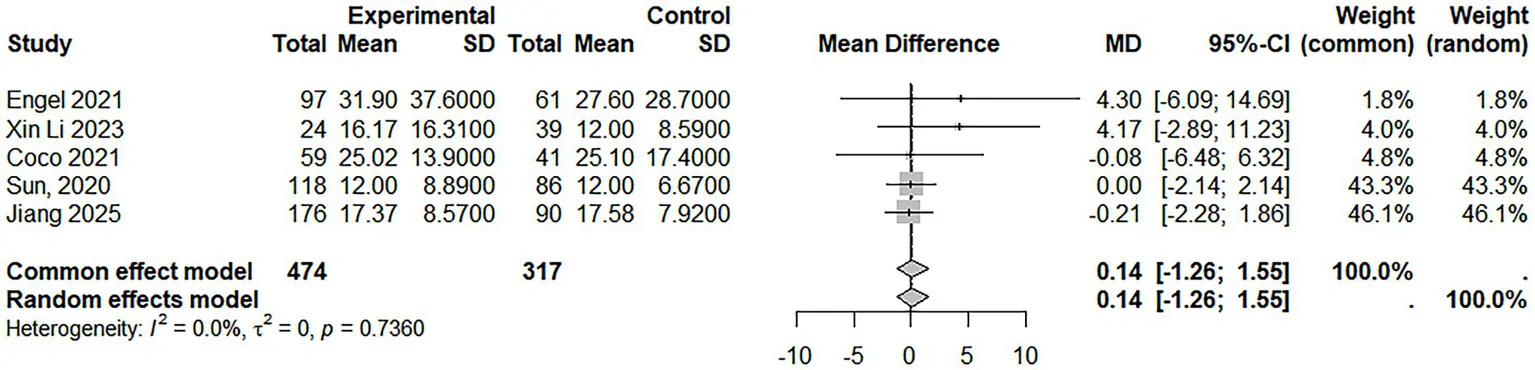
Differences in disease duration between ALS patients with and without sleep disorders.

In total, five studies were included, involving 474 ALS patients with sleep disorders and 317 ALS patients without sleep disorders. No substantial heterogeneity was identified among studies (*i*^2^ = 0%, *p* = 0.736). Thus, a fixed-effects model was applied for analysis. The findings uncovered that the difference in disease duration between individuals with and without sleep disorders was not statistically significant (MD = 0.14 months, 95% CI: −1.26–1.55) (Figure 6).

#### 3.3.6 Differences in BMI between individuals with and without sleep disorders

**Figure 7:**
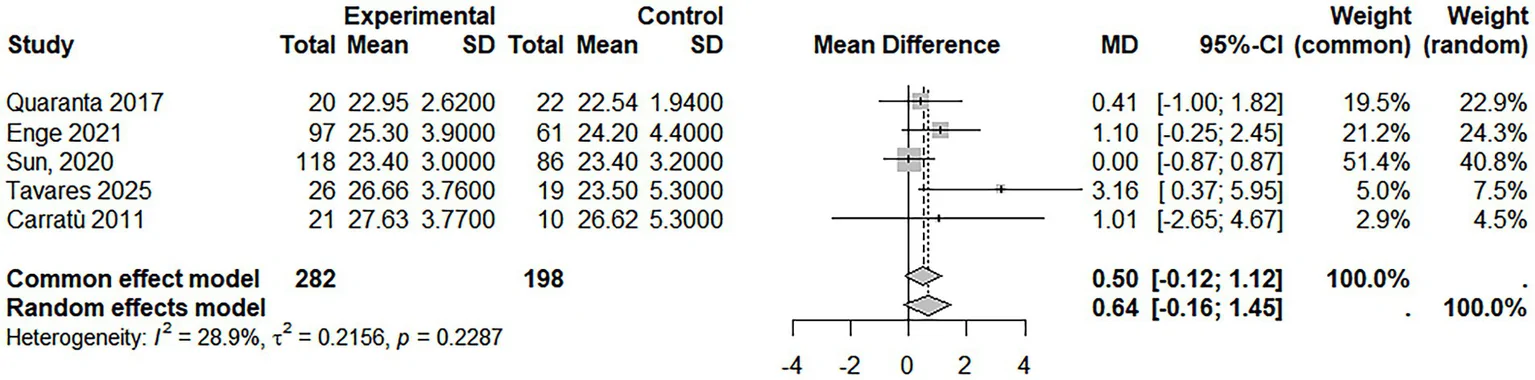
Differences in BMI between ALS patients with and without sleep disorders.

Totally five studies were incorporated, comprising 282 ALS patients with sleep disorders and 198 ALS patients without sleep disorders. The heterogeneity across studies was low (*i*^2^ = 28.9%, *p* = 0.2287), and a fixed-effects model was employed for analysis. The findings unveiled that the difference in BMI between individuals with and without sleep disorders was non-statistically significant (MD = 0.50 kg/m^2^, 95% CI: −0.12–1.12) (Figure 7).

#### 3.3.7 Comparison of sleep parameters between bulbar-onset and non-bulbar-onset ALS patients

**Figure 8:**
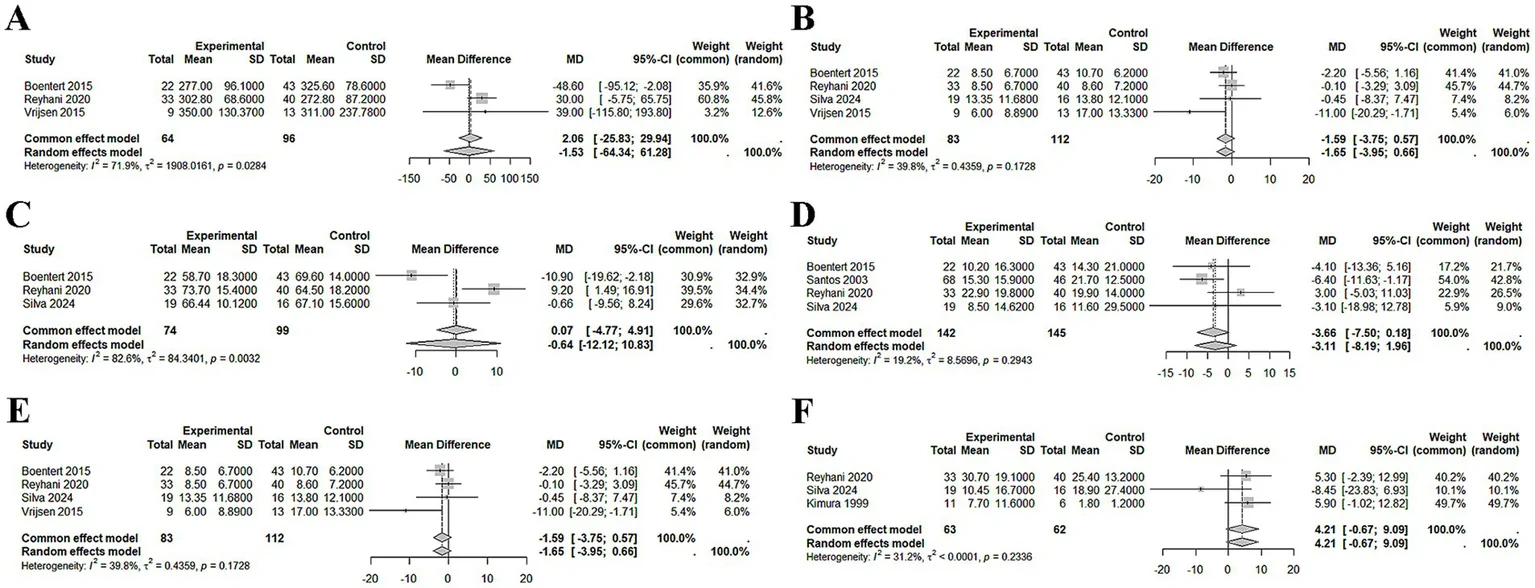
Differences in sleep parameters between bulbar-onset and non-bulbar-onset ALS patients with sleep disorders.

In the comparison of sleep parameters between bulbar-onset and non-bulbar-onset ALS patients (Figure 8), no statistically significant variations were identified for any of the indicators. Specifically, TST (MD = −1.53, 95% CI: −44.34 to 61.28, *i*^2^ = 71.9%) and SE (MD = −0.44, 95% CI: −12.12 to 10.83, *i*^2^ = 82.6%) showed no marked differences, with high heterogeneity. In addition, no substantial variations were found in REM, % (MD = −1.85, 95% CI: −3.95 to 0.26, *i*^2^ = 30.6%), AHI (MD = −3.11, 95% CI: −8.19 to 1.96, *i*^2^ = 19.2%), RDI (MD = 4.21, 95% CI: −0.67 to 9.09, *i*^2^ = 31.2%), or N3, % (MD = 1.25, 95% CI: −1.59 to 4.09, *i*^2^ = 0%) (Figure 8).

#### 3.3.8 Differences in the prevalence of sleep disorders according to the ICSD-3-TR classification in individuals with ALS

**Figure 9:**
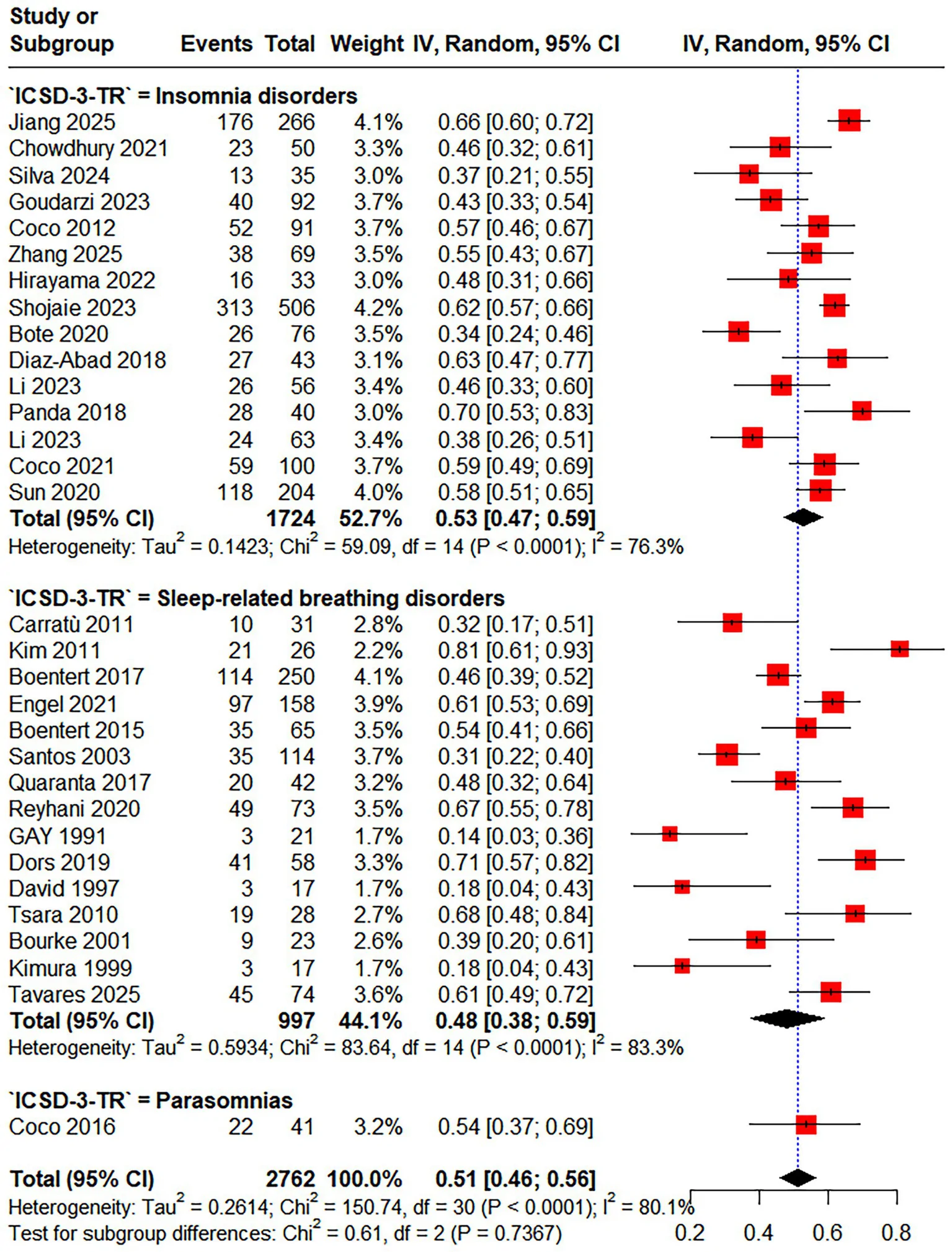
Differences in the prevalence of sleep disorders according to the ICSD-3-TR classification in individuals with ALS.

Based on the ICSD-3-TR classification, 15 studies involving 1,724 patients who were categorized as having insomnia disorders yielded prevalence estimates from individual studies ranging from 34 to 70%. The pooled prevalence was 53% (95% CI, 47–59%; *i*^2^ = 76.3%). 15 studies involving 997 patients who were categorized as having sleep-related breathing disorders reported prevalence estimates from individual studies ranging from 14 to 81%. The pooled prevalence was 48% (95% CI, 38–59%; *i*^2^ = 83.3%). One study involving 41 patients was classified into the parasomnia category and reported a prevalence of 54% (95% CI, 37–69%). The test for subgroup differences showed no statistically significant difference among the three ICSD-3-TR categories (*χ*^2^ = 0.61, df = 2, *p* = 0.7367). Because the parasomnia category was represented by only one study, its prevalence estimate should be interpreted cautiously (Figure 9).

#### 3.3.9 Exploratory subgroup analysis according to study-level reported sleep-related treatment status

**Figure 10:**
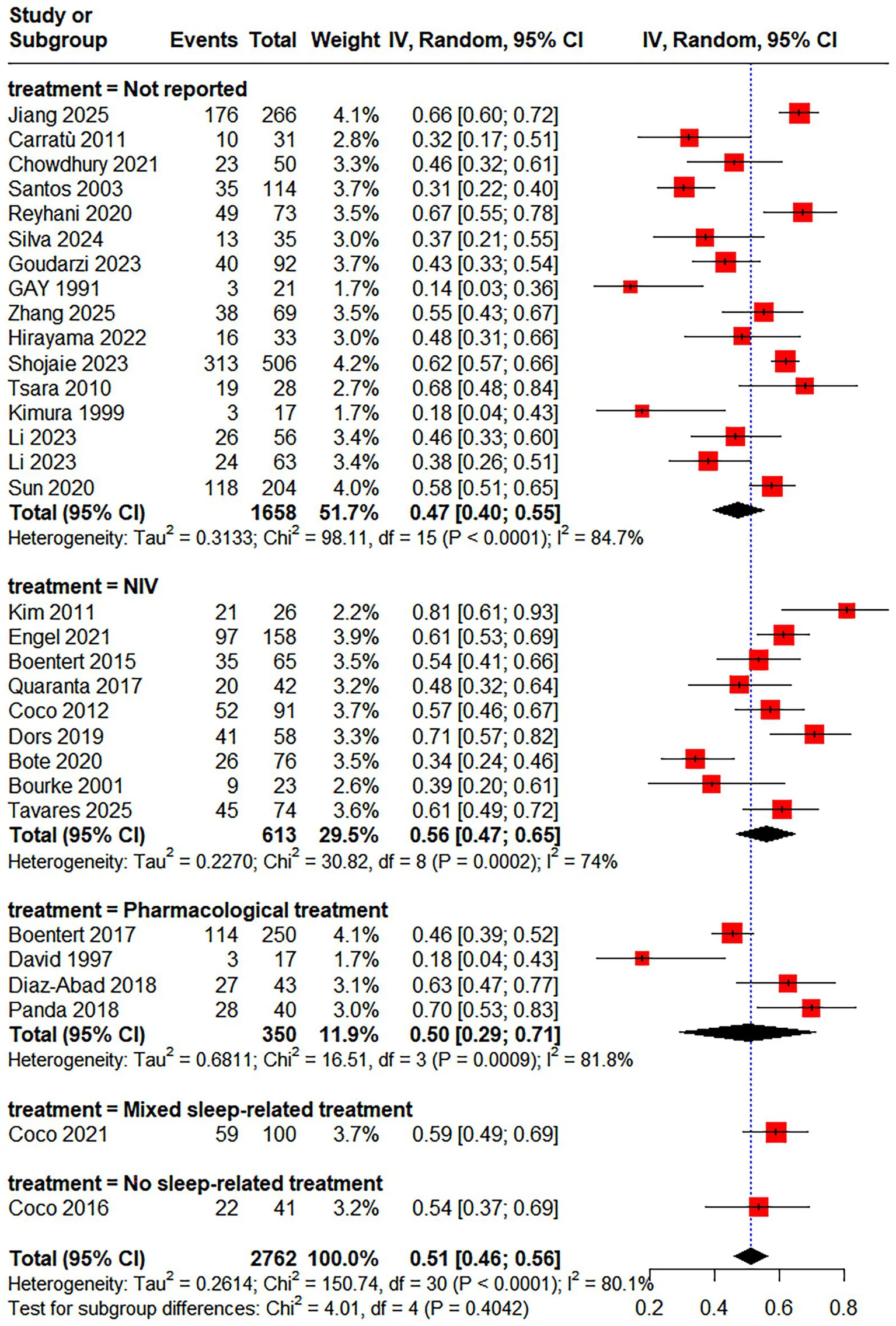
Exploratory subgroup analysis according to study-level reported sleep-related treatment status.

Studies were categorized according to reported sleep-related treatment status. 16 studies involving 1,658 patients did not report treatment information, with a pooled prevalence of 47% (95% CI 40–55%; *i*^2^ = 84.7%). Nine studies involving 613 patients reported the use of non-invasive ventilation, with a pooled prevalence of 56% (95% CI 47–65%; *i*^2^ = 74.0%). Four studies involving 350 patients reported pharmacological treatment, with a pooled prevalence of 50% (95% CI 29–71%; *i*^2^ = 81.8%). One study involving 100 patients reported mixed sleep-related treatment and had a prevalence of 59% (95% CI 49–69%), whereas one study involving 41 patients reported no sleep-related treatment and had a prevalence of 54% (95% CI 37–69%). No statistically significant difference was observed across categories of reported treatment status (*χ*^2^ = 4.01, df = 4, *p* = 0.4042). However, the reporting of an intervention at the study level did not necessarily indicate that all participants received that intervention, and individual-level treatment information was generally unavailable. Therefore, findings of this exploratory subgroup analysis should be interpreted cautiously and should not be considered evidence of treatment effects (Figure 10).

#### 3.3.10 Differences in the prevalence of sleep disorders according to the sleep assessment method in individuals with ALS

**Figure 11:**
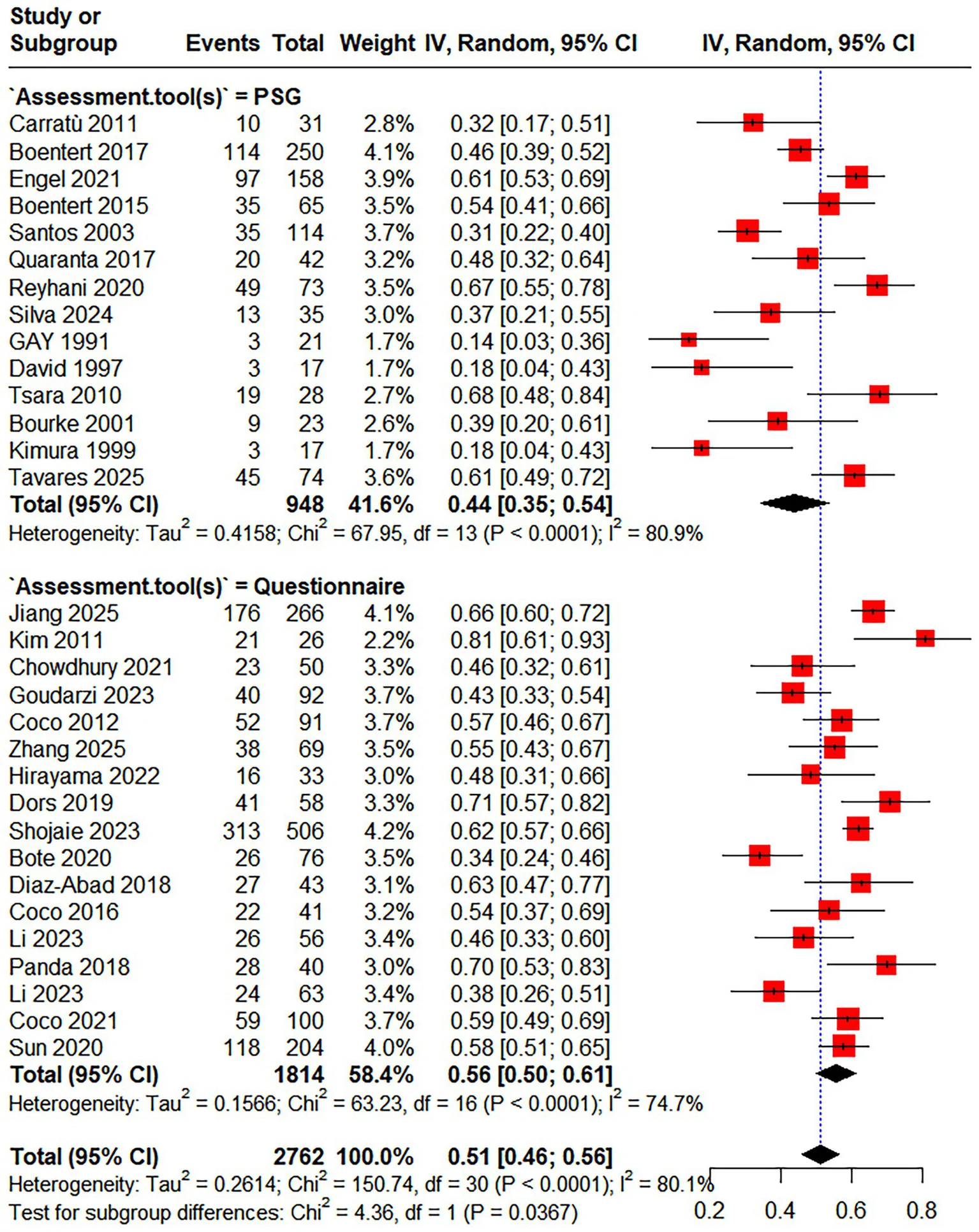
Differences in the prevalence of sleep disorders according to the sleep assessment method in individuals with ALS.

According to the assessment method corresponding to the prevalence estimate, 14 studies involving 948 patients used PSG-based assessments, with prevalence estimates from individual studies ranging from 14 to 68%. The pooled prevalence in this subgroup was 44% (95% CI 35–54%; *i*^2^ = 80.9%). 17 studies involving 1,814 patients used questionnaire-based assessments, with prevalence estimates from individual studies ranging from 34 to 81%, and the pooled prevalence was 56% (95% CI 50–61%; *i*^2^ = 74.7%). The difference between the two subgroups was statistically significant (*χ*^2^ = 4.36, df = 1, *p* = 0.0367). This indicated that questionnaire-based studies yielded a higher pooled prevalence than PSG-based studies (Figure 11).

## 4 Discussion

This systematic review and meta-analysis included 31 studies involving 2,762 individuals with ALS. The prevalence of sleep disorders and related clinical factors in ALS patients were comprehensively evaluated. The results revealed that sleep disturbances are highly prevalent among patients with ALS. This finding indicates that sleep dysfunction represents an important non-motor manifestation throughout the disease course. Thus, routine sleep assessment and early multidisciplinary management are needed in patients with ALS. Further subgroup analysis suggested that age might be a clinical factor linked to sleep disorders, whereas no substantial associations were observed for sex, disease duration, BMI, or site of onset. The prevalence estimates were broadly comparable across the ICSD-3-TR categories, although the estimate for parasomnias was based on a single study and should be interpreted cautiously.

The results of this study are generally consistent with previous research, further suggesting a high prevalence of sleep disorders in individuals with ALS (7, 10). Subgroup analysis showed that ALS patients with sleep disorders were substantially older than those without sleep disorders. Thus, age may be a potential contributing factor. Aging itself can lead to alterations in sleep architecture, like reduced slow-wave sleep, increased nocturnal awakenings, and decreased sleep continuity (32). Additionally, the prevalence of sleep-disordered breathing increases with age in the general population, and ALS-related respiratory muscle weakness and nocturnal hypoventilation may further amplify this effect (21, 33). Moreover, older ALS patients often have more comorbidities, functional limitations, and emotional issues, and these factors may collectively exacerbate sleep disorders (10, 13). Nevertheless, this finding should be interpreted with caution, as the effect of age may in part reflect disease severity, respiratory involvement, or comorbidity burden. Future large-sample prospective studies should be carried out to corroborate these results.

The occurrence of sleep disorders in individuals with ALS may be related to multiple pathophysiological mechanisms, which may provide possible explanations for the high prevalence observed in this study. First, as the disease progresses, the diaphragm and other respiratory muscles become progressively impaired, making individuals more prone to nocturnal hypoventilation and carbon dioxide retention during sleep, thereby potentially leading to frequent awakenings, sleep structure disruption, and impaired daytime function (8, 34). Second, in individuals with ALS, especially those with bulbar onset, reduced function of the pharyngeal and laryngeal muscles and decreased upper airway stability may increase the odds of obstructive sleep apnea and other sleep-related breathing disorders, which in turn are associated with nocturnal hypoxia, sleep fragmentation, and daytime sleepiness (16, 35, 36). Moreover, ALS-related neurodegenerative changes may involve key structures involved in sleep regulation, like the brainstem, thalamus, and hypothalamus, thereby disturbing normal sleep–wake rhythms (17, 18, 37). Previous research has suggested that hypothalamic atrophy and imbalance of the melanin-concentrating hormone (MCH)/orexin axis may contribute to the onset of sleep disorders, providing a possible explanation at the central mechanism level for sleep abnormalities in ALS patients (38, 39). Meanwhile, dysfunction of the glymphatic system may impair the clearance of metabolic waste and certain pathological proteins from the brain during sleep. In ALS, this process may be linked to TDP-43-related protein pathology, sleep disorders, and clinical severity (40, 41). In addition, concomitant symptoms like muscle cramps, pain, anxiety, and depression may further affect sleep continuity and quality (10, 23). Overall, sleep disorders in individuals with ALS are likely not caused by a single mechanism but rather result from the interplay of multiple factors, encompassing respiratory muscle weakness, upper airway dysfunction, involvement of central sleep-regulatory networks, dysfunction of the glymphatic system, and concomitant symptoms. These mechanisms may provide potential explanations for the findings. However, their specific pathways and relative contributions should be further validated in future high-quality prospective studies. Beyond their impact on quality of life, sleep disturbances should be considered within the multifactorial pathophysiological framework of ALS. Epidemiological studies have linked ALS susceptibility to potential environmental and occupational factors, including airborne heavy-metal pollution and military service, while recent analyses have challenged the applicability of a uniform multistep model to ALS pathogenesis (42–44). This broader risk framework supports longitudinal investigation of at-risk populations, in whom systematic sleep assessment may help characterize early non-motor alterations. Altered sleep microarchitecture has been identified in patients with early-stage ALS and presymptomatic carriers of ALS-associated mutations (45), while abnormal sleep duration and chronotype have been associated with incident ALS in a prospective cohort study (46). Nevertheless, current evidence is insufficient to establish sleep disturbances as specific prodromal biomarkers of ALS, and prospective longitudinal studies are required to clarify their temporal and pathophysiological significance. Moreover, sleep disorders frequently coexist in ALS and may exert synergistic effects rather than acting independently. The combination of sleep-disordered breathing, insomnia, RLS, periodic limb movements, and nocturnal hypoventilation may further impair sleep continuity, exacerbate neuroinflammation, and contribute to disease progression. These findings support a comprehensive assessment of coexisting sleep disorders in clinical practice (47). Importantly, similar observations have also been reported in other neurodegenerative disorders, particularly Alzheimer’s disease and frontotemporal dementia. In these conditions, sleep disturbances are increasingly recognized as early manifestations of neurodegeneration rather than merely secondary symptoms (48, 49). Shared mechanisms, including glymphatic dysfunction, neuroinflammation, and large-scale neural network disruption, further support the translational value of systematic sleep assessment for early disease recognition across neurodegenerative disorders (50).

The meta-analysis in the current study showed high heterogeneity among studies, suggesting considerable variations in methodology and subject characteristics across the included studies. First, the sleep assessment tools adopted varied across studies. Some used subjective scales (e.g., Pittsburgh sleep quality index (PSQI), Epworth sleepiness scale (ESS)), while others utilized objective assessments with PSG. Due to the limited agreement between subjective scales and objective sleep measures, the use of different assessment tools may result in differences in the estimated prevalence (51, 52). Second, the disease stage and respiratory function status of the included patients varied. Individuals at an advanced stage usually have more pronounced respiratory muscle weakness and are more prone to nocturnal hypoventilation and sleep-disordered breathing, thereby increasing the detection rate (34). Furthermore, differences in age, sex ratio, BMI, and site of onset among study subjects may also have a potential modulating effect on the occurrence of sleep disorders. Variations in healthcare resources, diagnostic strategies, and accessibility of sleep assessment tools across countries and regions, and some studies relying more on questionnaires than PSG, further reduced the comparability of the results (7, 52). Finally, inconsistencies in study design, sample size, and inclusion/exclusion criteria may also have influenced the pooled results. Overall, the high heterogeneity identified in this study may be attributable to multiple factors, like assessment methods, disease stage, population characteristics, regional differences, and study design. Future researchers should further standardize sleep assessment tools, patient stratification criteria, and study designs to enhance comparability of results and the reliability of meta-analysis conclusions.

The findings of the current study have important clinical implications. First, approximately half of ALS patients have sleep disorders. This result indicates that sleep problems are highly prevalent in this population and may still be underrecognized in routine clinical practice (7, 10). Therefore, integrating sleep assessment into standardized clinical management of ALS patients is recommended to facilitate early detection and intervention of non-motor symptoms. Second, the present study suggests that age may be an important factor related to sleep disorders. Although this relation needs to be further validated in large-sample prospective studies, from a clinical perspective, older patients could be prioritized for attention. Furthermore, although no statistically significant variations were identified in the comparison of various sleep parameters in this study, this result should be interpreted with caution, as it may be linked to the limited number of included studies. Previous evidence has shown that timely respiratory function assessment and appropriate use of non-invasive ventilation in individuals with ALS with nocturnal hypoventilation or sleep-related breathing abnormalities can improve sleep-related parameters. Some studies also suggest an association of this intervention with better survival outcomes (53, 54). In summary, systematic evaluation and individualized intervention for sleep disorders should be strengthened in the clinical management of ALS. In addition, particular attention should be paid to older patients and those with possible respiratory involvement, which may help optimize the long-term management of these patients.

The present study has several strengths. First, it systematically integrated the current evidence on sleep disorders in individuals with ALS. The current study encompassed 31 studies with 2,762 patients, representing a relatively large sample size, thereby enhancing the stability and reference value of the estimated results. Second, through multidimensional subgroup analyses including age, sex, BMI, disease duration, and site of onset, this study comprehensively evaluated potential associated factors, providing a more systematic evidence-based basis for understanding the occurrence of sleep disorders in ALS patients. Nevertheless, the present study also has some limitations. First, there was high heterogeneity among the included studies. Although subgroup and sensitivity analyses were carried out to appraise this heterogeneity, it may still affect the stability of the pooled results. Second, most of the included studies were observational, and some had small sample sizes, which may introduce selection, information, and confounding bias, thus affecting the internal validity of the results (55, 56). In addition, some subgroup analyses were based on a limited number of studies, and treatment information or assessment methods were incompletely reported in several studies. Consequently, these subgroup findings should be considered exploratory and require confirmation in future well-designed prospective studies. Furthermore, although most included studies applied internationally accepted ALS diagnostic criteria, a small number used different diagnostic standards or physician-confirmed diagnoses. Such differences may have contributed to clinical heterogeneity but could not be formally evaluated because of the limited number of studies within each diagnostic category. Moreover, upper motor neuron–predominant, lower motor neuron–predominant, and mixed upper and lower motor neuron phenotypes were insufficiently reported across the included studies, precluding phenotype-stratified analyses. Differences in sleep assessment methods across studies, including variations in the use of subjective scales and PSG, may also have affected the comparability of findings and the accuracy of prevalence estimates (51, 54). The included studies varied in sleep outcomes, assessment windows, instruments, and diagnostic thresholds. No prespecified hierarchy was established at the protocol stage for selecting among multiple outcomes, and “sleep disorder events” were not uniformly defined. Therefore, event counts relied on study-specific definitions and reported prevalence data, without retrospective harmonization of questionnaire or PSG thresholds. Because subjective assessments of recent sleep and single-night PSG measures are not directly comparable, the pooled estimate should be interpreted as a broad synthesis measure of study-defined sleep disorders or sleep-related abnormalities rather than as the prevalence estimate of a single uniformly defined condition. Differences in recall periods, PSG protocols, diagnostic cut-offs, and the potential first-night effect may have contributed to residual heterogeneity. Furthermore, the temporal relationship between sleep disturbances and ALS onset was generally unclear. Although most studies assessed sleep outcomes after ALS diagnosis or during the disease course, this observation does not necessarily establish that the sleep disturbances have developed secondary to ALS. Thus, pre-existing primary sleep disorders could not be reliably distinguished from those emerging during the course of ALS. Since the current study is primarily based on observational evidence, the analyses of associated factors should be interpreted as showing associations rather than causal conclusions. Thus, large-sample, multicenter, prospective studies are still required in the future, and sleep assessment tools, patient stratification criteria, and study designs should be further standardized, to improve the comparability of findings and the generalizability of conclusions.

## 5 Conclusion

This systematic review and meta-analysis show that the overall prevalence of sleep disorders in individuals with ALS is approximately 50%. This result indicates that sleep disorders are a common and easily overlooked non-motor symptom in ALS. Further analyses suggest that age may be associated with sleep disorders, whereas no substantial associations were observed for sex, BMI, disease duration, or site of onset. Subgroup analyses further indicated generally consistent findings across ICSD-defined sleep disorder categories and treatment subgroups, whereas questionnaire-based assessments identified a higher prevalence than PSG-based assessments. Thus, it is important to consider differences between subjective and objective measures when interpreting sleep outcomes. In summary, sleep disorders have important clinical implications in ALS patients, and routine screening, dynamic assessment, and individualized intervention should be strengthened in clinical management. Future large-sample, multicenter, prospective studies are still needed to further clarify the mechanisms underlying sleep disorders in individuals with ALS and their relationship with disease progression and prognosis.

## Data Availability

The original contributions presented in the study are included in the article/Supplementary material, further inquiries can be directed to the corresponding author.
