# Supplementary material for: Prevalence of and associated factors for sleep disorders in patients with amyotrophic lateral sclerosis: a systematic review and meta-analysis
Source: Front Neurol. 2026 Aug 31;17:1889217. doi: 10.3389/fneur.2026.1889217 (PMC13572260; doi:10.3389/fneur.2026.1889217)
Supplement: Supplementary file 1 [file Supplementary_file_1.DOCX]

**Table S1.** Search Histories of Each Database.

| Databases | Search number | Search Details | Results | Date |
| --- | --- | --- | --- | --- |
| PubMed | 1 | Amyotrophic Lateral Sclerosis[MeSH Terms] | 26,322 | 10-Jan-2026 |
|  | 2 | ALS[Title/Abstract] OR Amyotrophic Lateral Sclerosis[Title/Abstract] OR Lou Gehrig's Disease[Title/Abstract] OR Lou Gehrig Disease[Title/Abstract] OR Charcot Disease[Title/Abstract] OR Motor Neuron Disease[Title/Abstract] OR Motor Neurone Disease[Title/Abstract] OR MND[Title/Abstract] OR Guam Disease[Title/Abstract] OR Guam Form of Amyotrophic Lateral Sclerosis[Title/Abstract] OR ALS Dementia[Title/Abstract] OR Dementia with Amyotrophic Lateral Sclerosis[Title/Abstract] OR ALS-FTD[Title/Abstract] OR FTD-ALS[Title/Abstract] | 56,018 | 10-Jan-2026 |
|  | 3 | Sleep[MeSH Terms] | 110,969 | 10-Jan-2026 |
|  | 4 | sleep[Title/Abstract] OR sleeper[Title/Abstract] OR sleeping[Title/Abstract] OR Dyssomnias[Title/Abstract] OR insomnia[Title/Abstract] OR circadian rhythm disorder[Title/Abstract] OR Dyssomnia[Title/Abstract] | 294,566 | 10-Jan-2026 |
|  | 5 | (Amyotrophic Lateral Sclerosis[MeSH Terms]) OR (ALS[Title/Abstract] OR Amyotrophic Lateral Sclerosis[Title/Abstract] OR Lou Gehrig's Disease[Title/Abstract] OR Lou Gehrig Disease[Title/Abstract] OR Charcot Disease[Title/Abstract] OR Motor Neuron Disease[Title/Abstract] OR Motor Neurone Disease[Title/Abstract] OR MND[Title/Abstract] OR Guam Disease[Title/Abstract] OR Guam Form of Amyotrophic Lateral Sclerosis[Title/Abstract] OR ALS Dementia[Title/Abstract] OR Dementia with Amyotrophic Lateral Sclerosis[Title/Abstract] OR ALS-FTD[Title/Abstract] OR FTD-ALS[Title/Abstract]) | 58,128 | 10-Jan-2026 |
|  | 6 | (Sleep[MeSH Terms]) OR (sleep[Title/Abstract] OR sleeper[Title/Abstract] OR sleeping[Title/Abstract] OR Dyssomnias[Title/Abstract] OR insomnia[Title/Abstract] OR circadian rhythm disorder[Title/Abstract] OR Dyssomnia[Title/Abstract]) | 312,903 | 10-Jan-2026 |
|  | 7 | ((Amyotrophic Lateral Sclerosis[MeSH Terms]) OR (ALS[Title/Abstract] OR Amyotrophic Lateral Sclerosis[Title/Abstract] OR Lou Gehrig's Disease[Title/Abstract] OR Lou Gehrig Disease[Title/Abstract] OR Charcot Disease[Title/Abstract] OR Motor Neuron Disease[Title/Abstract] OR Motor Neurone Disease[Title/Abstract] OR MND[Title/Abstract] OR Guam Disease[Title/Abstract] OR Guam Form of Amyotrophic Lateral Sclerosis[Title/Abstract] OR ALS Dementia[Title/Abstract] OR Dementia with Amyotrophic Lateral Sclerosis[Title/Abstract] OR ALS-FTD[Title/Abstract] OR FTD-ALS[Title/Abstract])) AND ((Sleep[MeSH Terms]) OR (sleep[Title/Abstract] OR sleeper[Title/Abstract] OR sleeping[Title/Abstract] OR Dyssomnias[Title/Abstract] OR insomnia[Title/Abstract] OR circadian rhythm disorder[Title/Abstract] OR Dyssomnia[Title/Abstract])) | 585 | 10-Jan-2026 |
| Embase | 1 | 'amyotrophic lateral sclerosis'/exp | 59839 | 10-Jan-2026 |
|  | 2 | 'sleep'/exp | 363548 | 10-Jan-2026 |
|  | 3 | als:ab,ti OR 'amyotrophic lateral sclerosis':ab,ti OR 'lou gehrigs disease':ab,ti OR 'lou gehrig disease':ab,ti OR 'charcot disease':ab,ti OR 'motor neuron disease':ab,ti OR 'motor neurone disease':ab,ti OR mnd:ab,ti OR 'guam disease':ab,ti OR 'guam form of amyotrophic lateral sclerosis':ab,ti OR 'als dementia':ab,ti OR 'dementia with amyotrophic lateral sclerosis':ab,ti OR 'als ftd':ab,ti OR 'ftd als':ab,ti | 75374 | 10-Jan-2026 |
|  | 4 | sleep:ab,ti OR sleeper:ab,ti OR sleeping:ab,ti OR dyssomnias:ab,ti OR insomnia:ab,ti OR 'circadian rhythm disorder':ab,ti OR dyssomnia:ab,ti | 460572 | 10-Jan-2026 |
|  | 5 | #1 OR #3 | 89006 | 10-Jan-2026 |
|  | 6 | #2 OR #4 | 576361 | 10-Jan-2026 |
|  | 7 | #5 AND #6 | 1584 | 10-Jan-2026 |
| Cochrane | 1 | MeSH descriptor: [Amyotrophic Lateral Sclerosis] explode all trees | 929 | 10-Jan-2026 |
|  | 2 | (ALS OR Amyotrophic Lateral Sclerosis OR Lou Gehrig's Disease OR Lou Gehrig Disease OR Charcot Disease OR Motor Neuron Disease OR Motor Neurone Disease OR MND OR Guam Disease OR Guam Form of Amyotrophic Lateral Sclerosis OR ALS Dementia OR Dementia with Amyotrophic Lateral Sclerosis OR ALS-FTD OR FTD-ALS):ti,ab,kw | 3854 | 10-Jan-2026 |
|  | 3 | #1 OR #2 | 3854 | 10-Jan-2026 |
|  | 4 | MeSH descriptor: [Sleep] explode all trees | 9392 | 10-Jan-2026 |
|  | 5 | (sleep OR sleeper OR sleeping OR Dyssomnias OR insomnia OR circadian rhythm disorder OR Dyssomnia):ti,ab,kw | 72080 | 10-Jan-2026 |
|  | 6 | #4 OR #5 | 72240 | 10-Jan-2026 |
|  | 7 | #3 AND #6 | 185 | 10-Jan-2026 |
| Web of Science | 1 | ALS OR Amyotrophic Lateral Sclerosis OR Lou Gehrig's Disease OR Lou Gehrig Disease OR Charcot Disease OR Motor Neuron Disease OR Motor Neurone Disease OR MND OR Guam Disease OR Guam Form of Amyotrophic Lateral Sclerosis OR ALS Dementia OR Dementia with Amyotrophic Lateral Sclerosis OR ALS-FTD OR FTD-ALS (Topic) and sleep OR sleeper OR sleeping OR Dyssomnias OR insomnia OR circadian rhythm disorder OR Dyssomnia (Topic) | 1585 | 10-Jan-2026 |

**Table S2. Additional characteristics of the included studies.**

| Study | ALS diagnostic criteria | ICSD-3-TR | Assessment tool(s) | Reported sleep-related treatment (type and proportion) | Timing relative to ALS onset |
| --- | --- | --- | --- | --- | --- |
| Jiang 2025 | Gold Coast Criteria | Insomnia Disorder | Questionnaire | Not reported | Not reported |
| Carratù 2011 | El Escorial criteria | Sleep-related breathing disorders | PSG | Not reported | Not reported |
| Kim 2011 | El Escorial criteria | Sleep-related breathing disorders | Questionnaire | NIV（100%） | Not reported |
| Chowdhury 2021 | Revised El Escorial criteria | Insomnia Disorder | Questionnaire | Not reported | Not reported |
| Boentert 2017 | Revised El Escorial criteria | Sleep-related breathing disorders | PSG | Pharmacological treatment(4%) | Not reported |
| Engel 2021 | Revised El Escorial criteria | Sleep-related breathing disorders | PSG | NIV(100%) | Not reported |
| Boentert 2015 | Revised El Escorial criteria | Sleep-related breathing disorders | PSG | NIV(100%) | Not reported |
| Santos 2003 | El Escorial criteria | Sleep-related breathing disorders | PSG | Not reported | Not reported |
| Quaranta 2017 | Not specified (confirmed ALS diagnosis) | Sleep-related breathing disorders | PSG | NIV(100%) | Not reported |
| Reyhani 2020 | Revised El Escorial criteria | Sleep-related breathing disorders | PSG | Not reported | Not reported |
| Silva 2024 | Not specified (confirmed ALS diagnosis) | Insomnia disorders | PSG | Not reported | Not reported |
| Goudarzi 2023 | Revised El Escorial criteria | Insomnia disorders | Questionnaire | Not reported | Not reported |
| Gay 1991 | Not specified (confirmed ALS diagnosis) | Sleep-related breathing disorders | PSG | Not reported | Not reported |
| Coco 2012 | Revised El Escorial criteria | Insomnia disorders | Questionnaire | NIV(23%) | Not reported |
| Zhang 2025 | Revised El Escorial criteria | Insomnia disorders | Questionnaire | Not reported | Not reported |
| Hirayama 2022 | Not specified (confirmed ALS diagnosis) | Insomnia disorders | Questionnaire | Not reported | Not reported |
| Dorst 2019 | Revised El Escorial criteria | Sleep-related breathing disorders | Questionnaire | NIV (100%) | Not reported |
| Shojaie 2023 | Not specified (confirmed ALS diagnosis) | Insomnia disorder | Questionnaire | Not reported | Not reported |
| Bote 2020 | El Escorial criteria | Insomnia disorders | Questionnaire | NIV(100%) | Not reported |
| David 1997 | Not reported | Sleep-related breathing disorders | PSG | Pharmacological treatment(33%) | Not reported |
| Diaz-Abad 2018 | Revised El Escorial criteria | Insomnia disorders | Questionnaire | Pharmacological treatment(19%) | Not reported |
| Coco 2016 | Revised El Escorial criteria | Parasomnias | Questionnaire | No sleep-related treatment | Developed after ALS onset |
| Tsara 2010 | Not reported | Sleep-related breathing disorders | PSG | Not reported | Not reported |
| Bourke 2001 | El Escorial criteria | Sleep-related breathing disorders | PSG | NIV(11%) | Not reported |
| Kimura 1999 | El Escorial criteria | Sleep-related breathing disorders | PSG | Not reported | Not reported |
| Li 2023a | Revised El Escorial criteria | Insomnia disorders | Questionnaire | Not reported | Not reported |
| Panda 2018 | El Escorial criteria | Insomnia disorders | Questionnaire | Pharmacological treatment(11%) | Not reported |
| Li 2023b | Revised El Escorial criteria | Insomnia disorders | Questionnaire | Not reported | Not reported |
| Coco 2021 | Revised El Escorial criteria | Insomnia disorders | Questionnaire | Mixed sleep-related treatment:Pharmacological treatment(22%),NIV(36%) | Developed after ALS onset |
| Sun 2020 | Awaji criteria | Insomnia disorders | Questionnaire | Not reported | Not reported |
| Tavares 2025 | Not specified (confirmed ALS diagnosis) | Sleep-related breathing disorders | PSG | NIV(100%) | Not reported |

**Note:** Treatment information was extracted as reported at the study level and did not necessarily indicate that all participants received the reported intervention.

**Abbreviations:** PSG: Polysomnography; NIV: Non-invasive ventilation; ICSD-3-TR: International Classification of Sleep Disorders, Third Edition, Text Revision.

**Table S3. Quality Assessment Tool for Observational Cohort and Cross-Sectional Studies.**

| Study | Q1 | Q2 | Q3 | Q4 | Q5 | Q6 | Q7 | Q8 | Q9 | Q10 | Q11 | Q12 | Q13 | Q14 | Grade |
| --- | --- | --- | --- | --- | --- | --- | --- | --- | --- | --- | --- | --- | --- | --- | --- |
| Jiang 2025 | √ | √ | √ | √ | × | NA | NA | √ | √ | NA | √ | × | NA | √ | Good |
| Carratù 2011 | √ | √ | √ | √ | × | × | × | √ | √ | × | √ | × | NA | √ | Fair |
| Kim 2011 | √ | √ | √ | √ | × | √ | √ | √ | √ | × | √ | × | √ | × | Good |
| Chowdhury 2021 | √ | √ | √ | √ | × | NA | NA | √ | √ | NA | √ | × | NA | √ | Fair |
| Boentert 2017 | √ | √ | √ | √ | × | NA | NA | √ | √ | NA | √ | × | NA | √ | Good |
| Engel 2021 | √ | √ | √ | √ | × | √ | √ | √ | √ | × | √ | NA | √ | √ | Good |
| Boentert 2015 | √ | √ | √ | √ | × | √ | √ | NA | √ | √ | √ | × | × | √ | Good |
| Santos 2003 | √ | NR | NA | √ | × | NA | NA | √ | √ | NA | √ | × | NA | √ | Fair |
| Quaranta 2017 | √ | √ | √ | √ | × | √ | √ | NA | √ | × | √ | × | √ | √ | Good |
| Reyhani 2020 | √ | √ | √ | √ | × | √ | √ | √ | √ | × | √ | NR | √ | √ | Good |
| Silva 2024 | √ | √ | √ | √ | × | √ | √ | √ | √ | × | √ | NR | √ | √ | Fair |
| Goudarzi 2023 | √ | √ | NA | √ | × | NA | NA | √ | √ | NA | √ | × | NA | √ | Good |
| GAY 1991 | √ | NR | √ | √ | × | √ | √ | √ | √ | × | √ | × | √ | NR | Fair |
| Coco 2012 | √ | √ | NA | √ | × | NA | NA | √ | √ | NA | √ | × | NA | √ | Fair |
| Zhang 2025 | √ | √ | NA | √ | × | NA | NA | √ | √ | NA | √ | × | NA | √ | Good |
| Hirayama 2022 | √ | NR | NA | × | × | NA | NA | √ | NR | NA | × | × | NA | × | Poor |
| Dors 2019 | √ | √ | √ | √ | × | √ | √ | √ | √ | √ | √ | × | NR | NR | Good |
| Shojaie 2023 | √ | NR | NA | × | × | NA | NA | NA | × | NA | × | × | NA | × | Fair |
| Bote 2020 | √ | √ | √ | √ | × | √ | √ | √ | √ | × | √ | × | √ | NR | Good |
| David 1997 | √ | √ | NA | NR | × | NA | NA | √ | √ | NA | √ | × | NA | × | Fair |
| Diaz-Abad 2018 | √ | √ | NA | √ | × | NA | NA | √ | √ | NA | √ | × | NA | √ | Good |
| Coco 2016 | √ | √ | NA | √ | × | NA | NA | √ | √ | NA | √ | × | NA | √ | Good |
| Tsara V 2010 | √ | NR | NA | NR | × | NA | NA | √ | √ | NA | √ | × | NA | × | Fair |
| Bourke 2001 | √ | √ | NA | √ | × | NA | NA | √ | √ | NA | √ | × | NA | √ | Good |
| Kimura 1999 | √ | √ | NA | √ | × | NA | NA | √ | √ | NA | √ | × | NA | × | Fair |
| Li 2023a | √ | √ | NA | √ | × | NA | NA | √ | √ | NA | √ | × | NA | √ | Good |
| Panda 2018 | √ | √ | NA | √ | × | NA | NA | √ | √ | NA | √ | × | NA | √ | Good |
| Li 2023b | √ | √ | √ | √ | × | √ | √ | √ | √ | √ | √ | × | √ | √ | Good |
| Tavares 2025 | √ | √ | NA | √ | × | NA | NA | √ | √ | NA | √ | × | NA | × | Fair |

Q1:Was the research question or objective in this paper clearly stated? Q2:Was the study population clearly specified and defined? Q3:Was the participation rate of eligible persons at least 50%? Q4:Were all the subjects selected or recruited from the same or similar populations (including the same time period)? Were inclusion and exclusion criteria for being in the study prespecified and applied uniformly to all participants? Q5:Was a sample size justification, power description, or variance and effect estimates provided? Q6:For the analyses in this paper, were the exposure(s) of interest measured prior to the outcome(s) being measured? Q7:Was the timeframe sufficient so that one could reasonably expect to see an association between exposure and outcome if it existed? Q8:For exposures that can vary in amount or level, did the study examine different levels of the exposure as related to the outcome (e.g., categories of exposure, or exposure measured as continuous variable)? Q9:Were the exposure measures (independent variables) clearly defined, valid, reliable, and implemented consistently across all study participants? Q10:Was the exposure(s) assessed more than once over time? Q11:Were the outcome measures (dependent variables) clearly defined, valid, reliable, and implemented consistently across all study participants? Q12:Were the outcome assessors blinded to the exposure status of participants? Q13:Was loss to follow-up after baseline 20% or less? Q14:Were key potential confounding variables measured and adjusted statistically for their impact on the relationship between exposure(s) and outcome(s)?

**Table S4. Quality Assessment Tool for Case-Control Studies.**

| Study | Q1 | Q2 | Q3 | Q4 | Q5 | Q6 | Q7 | Q8 | Q9 | Q10 | Q11 | Q12 | Grade |
| --- | --- | --- | --- | --- | --- | --- | --- | --- | --- | --- | --- | --- | --- |
| Coco 2021 | √ | √ | NR | × | √ | √ | √ | × | √ | × | √ | × | Fair |
| Sun 2020 | √ | √ | √ | × | √ | √ | √ | × | √ | × | √ | × | Dood |

Q1:Was the research question or objective in this paper clearly stated and appropriate? Q2:Was the study population clearly specified and defined? Q3:Did the authors include a sample size justification? Q4:Were controls selected or recruited from the same or similar population that gave rise to the cases (including the same timeframe)? Q5:Were the definitions, inclusion and exclusion criteria, algorithms or processes used to identify or select cases and controls valid, reliable, and implemented consistently across all study participants? Q6: Were the cases clearly defined and differentiated from controls? Q7:If less than 100 percent of eligible cases and/or controls were selected for the study, were the cases and/or controls randomly selected from those eligible? Q8: Was there use of concurrent controls? Q9:Were the investigators able to confirm that the exposure/risk occurred prior to the development of the condition or event that defined a participant as a case? Q10:Were the measures of exposure/risk clearly defined, valid, reliable, and implemented consistently (including the same time period) across all study participants? Q11:Were the assessors of exposure/risk blinded to the case or control status of participants? Q12:Were key potential confounding variables measured and adjusted statistically in the analyses? If matching was used, did the investigators account for matching during study analysis?

**Figure S1.** Sensitivity analysis.
